# Bio-inspired cofacial Fe porphyrin dimers for efficient electrocatalytic CO_2_ to CO conversion: Overpotential tuning by substituents at the porphyrin rings

**DOI:** 10.1038/srep24533

**Published:** 2016-04-18

**Authors:** Zaki N. Zahran, Eman A. Mohamed, Yoshinori Naruta

**Affiliations:** 1 Institute for Science and Technology Research, Centre for Chemical Energy Conversion, Chubu University, Kasugai 487-8501, Japan; 2 Faculty of Science, Tanta University, Tanta, Egypt

## Abstract

Efficient reduction of CO_2_ into useful carbon resources particularly CO is an essential reaction for developing alternate sources of fuels and for reducing the greenhouse effect of CO_2_. The binuclear Ni, Fe*−*containing carbon monoxide dehydrogenase (CODHs) efficiently catalyzes the reduction of CO_2_ to CO. The location of Ni and Fe at proper positions allows their cooperation for CO_2_ to CO conversion through a push−pull mechanism. Bio−inspired from CODHs, we used several cofacial porphyrin dimers with different substituents as suitable ligands for holding two Fe ions with suitable Fe−Fe separation distance to efficiently and selectively promote CO_2_ to CO conversion with high turnover frequencies, TOFs. The substituents on the porphyrin rings greatly affect the catalysis process. By introducing electron-withdrawing/-donating groups, e.g. electron-withdrawing perfluorophenyl, at all meso positions of the porphyrin rings, the catalysis overpotential, *η* was minimized by ≈0.3 V compared to that obtained by introducing electron-donating mesityl groups. The Fe porphyrin dimers among reported catalysts are the most efficient ones for CO_2_ to CO conversion. Control experiments indicate that the high performance of the current CO_2_ to CO conversion catalysts is due to the presence of binuclear Fe centers at suitable Fe−Fe separation distance.

The efficient reduction of CO_2_ into useful carbon resources particularly CO is an essential reaction to overcome the limited supply of fossil fuels and the greenhouse effect of CO_2_^1^,^2^,^3^. The CO_2_ to CO reduction is, for example, useful for generating syngas (CO + H_2_), which can be used to generate a wide variety of fuels with the current Fischer-Tropsch technologies^4^. Moreover, CO is a useful resource in methanol synthesis^5^ and in hydroformylation^6^. Plenty of homogeneous or heterogeneous CO_2_ to CO conversion catalysts have been reported. These include precious metal- (e.g. Re^7^,^8^,^9^,^10^,^11^, Ru^12^,^13^,^14^,^15^, Ir^16^, Au^17^,^18^, etc.) or non-precious metal- (e.g. Fe^19^,^20^,^21^,^22^,^23^, Ni^24^,^25^,^26^, Mn^27^,^28^ etc.) based catalysts. For large-scale fuel production, the development of base metal catalysts is essential^29^.

As base metal catalysts, Fe porphyrin monomers have been reported to efficiently catalyze the electrochemical reduction of CO_2_ to CO in the presence of external or localized phenol as a weak proton donor to act as a proton relay during CO_2_ to CO conversion. The electro-generated Fe^0^ porphyrin undergoes a concerted proton-electron transfer bond cleavage (CPETBC: electron transfer from the central Fe atom concerted with proton transfer and breaking of one C-O bond) rate-determining step to form an Fe−CO species that undergoes further reduction to release CO^19^,^20^,^21^,^22^,^23^.

In biological systems, several anaerobic bacteria and archaea utilize Ni, Fe*−*containing carbon monoxide dehydrogenases (CODHs) with a [NiFe_4_S_4_] cluster at the active site as highly efficient catalyst for CO_2_ to CO conversion with high turnover frequency, TOF (12 s^−1^) at a low overpotential, *η* < 100 mV^30^,^31^,^32^,^33^,^34^,^35^,^36^. Binding to Ni and Fe activates CO_2_ where Ni acts as a Lewis base to transfer a net electron to the antibonding lowest unoccupied molecular orbital of CO_2_ that increases the negative partial charges at the oxygen atoms which are stabilized by binding to a Lewis acid Fe center^30^,^31^,^32^,^33^,^34^,^35^,^36^. The biological CO_2_ to CO conversion indicates the importance of the binuclear metal ion centers with suitable separation distance in designing highly efficient catalysts for CO_2_ to CO conversion. Indeed, CO_2_ to CO conversion have been demonstrated with several binuclear- (e.g. Ni_2_^37^,^38^,^39^, Cu_2_^40^, Ir_2_^41^, and Pd_2_^42^) and polynuclear- (e.g. Ni_3_^43^,^44^, Fe_4_^45^, and iron-sulfur clusters; [Fe_4_S_4_(SR)_4_]^2−^, Ni−Fe_4_S_4_ and Co−Fe_4_S_4_^46^,^47^,^48^) catalysts, however, with low TOF and/or high *η*-values.

Inspired from the Ni, Fe−containing CODHs, we recently reported a cofacial Fe tetraphenylporphyrin dimer, *o*-Fe_2_DTPP having binuclear Fe centers at a suitable Fe−Fe separation distance that efficiently and selectively catalyzes the electrochemical CO_2_ to CO conversion in a DMF/10% H_2_O solution with high Faradic efficiency (95%) and TOF (4,300 s^−1^) without use of any acids. However, its reaction showed a relatively high overpotential, *η* = 0.66 V. Control experiments with the mononuclear Fe porphyrin monomer, FeTPP and the 1,3-phenylene bridged binuclear Fe porphyrin dimer, *m*-Fe_2_DTPP indicate the importance of the binuclear Fe centers and the Fe−Fe separation distance for the CO_2_ to CO conversion^49^. Here we introduced electron-donating and electron-withdrawing substituents to the peripheral porphyrin rings (Fig. 1) that tuned *η* and the activity of the catalytic process. We also performed control experiments with Fe porphyrin monomers (Fig. 1) that show low activity for CO_2_ reduction compared to that obtained with the binuclear Fe porphyrin dimers that clearly demonstrate the importance of the binuclear metal centers for the high activity and stability in designing CO_2_ to CO conversion molecular catalysts. Benchmarking with other catalysts, the binuclear Fe porphyrin dimers are, to the best of our knowledge, the most efficient and stable homogeneous molecular catalysts for CO_2_ to CO conversion at present.

## Results

Six porphyrin dimer ligands linked by a 1,2-phenylene bridge and bearing different substituents at the porphyrin rings have been prepared according to stepwise methods outlined in Scheme S1 (SI). Fe was then inserted into the porphyrin cavities by similar reported procedures^49^. Typically excess FeBr_2_ was refluxed with the porphyrin dimer ligand in DMF overnight under Ar. The compounds were purified by column chromatography and characterized with ordinary spectroscopic methods including UV-vis., ^1^HNMR, and mass spectroscopy (Figure S1, SI). For control experiments, we also prepared their corresponding Fe porphyrin monomers.

In a DMF/0.1 M ^*n*^But_4_NPF_6_ (^*n*^But_4_NPF_6_ = tetra-*n*-butylammonium hexafluorophosphate) solution saturated with Ar, the Fe porphyrin dimers (0.5 mM) and their corresponding Fe porphyrin monomers (1 mM) depict cyclic voltammetric (CV) behaviors shown in Figure S2 (SI) at a 50 mV/s scan rate. The Fe porphyrin monomers show, as previously reported^34^,^35^,^36^,^37^,^38^, three successive reversible 1e^−^ reductions/oxidations of the Fe centers, *i.e*. Fe^III/II^, Fe^II/1^ and Fe^I/0^ at standard redox potentials donated as *E*^0^(1), *E*^0^(2), and *E*^0^(3), respectively. The Fe porphyrin dimers, on the other hand, show three successive 2e^−^ reductions/oxidations of the two Fe centers represented by equations (1)~(6) at standard redox potentials donated as *E*^0^(1), *E*^0^(2), and *E*^0^(3) for 2Fe^III/II^, 2Fe^II/1^ and 2Fe^I/0^, respectively. In some cases, the 2e^−^ reductions/oxidations process of the dimers are divided into two successive 1e^−^ reductions/oxidations with standard redox potentials donated as, for example, *E*^0^(1a) and *E*^0^(1b). The number of electrons was confirmed by controlled-potential electrolysis carried out at 100 mV negative potentials of the peak potentials. Table S1 (SI) summarizes the standard redox potentials, *E*^0^, of the Fe porphyrin dimers and their corresponding Fe porphyrin monomers *vs.* NHE (hereafter, all potentials are indicated against NHE except as noted). The standard redox potential of the 2Fe^III/II^ reductions/oxidations of the dimers, *E*^0^(1) occur at significant negative potentials relative to that of the monomer possibly due to strong Cl^−^ binding/association to the Fe centers between the two cofacial porphyrin bi-layers^49^. The peak currents of the Fe porphyrin dimers are significantly smaller than that of the corresponding Fe porphyrin monomers. This is consistent with their small diffusion coefficients resulting from their large molecular sizes relative to their corresponding Fe porphyrin monomers^50^. The reduction peak currents of the Fe porphyrin dimers change linearly with the square root of the scan rate,*υ*^1/2^ indicating diffusion controlled electron transfer processes^50^. The standard redox potentials, *E*^0^, reflect the different electronic environment around the Fe centers. For example, *E*^0^s are shifted to more positive potentials as the electron-withdrawing properties of the substituents on the peripheral porphyrin rings increase. Also, a clear separation among *E*^0^(1a) and *E*^0^(1b), *E*^0^(2a) and *E*^0^(2b), and *E*^0^(3a) and *E*^0^(3b) couples were observed for the Fe_2_TPFPP-TMP dimer that has a significant difference in the electronic environment around the two Fe centers.


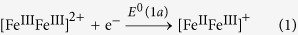



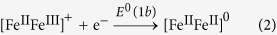



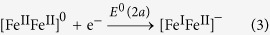



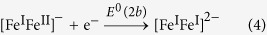



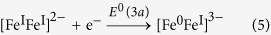



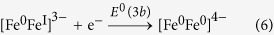


Under CO_2_, the CV behaviors of the Fe porphyrin dimers (0.5 mM) are depicted in Fig. 2 (red lines). The reversible 2Fe^III^/2Fe^II^ redox couple observed under Ar is replaced with a new reduction peak due, as we previously reported^49^, to the dissociation of the Cl^−^ and the coordination of the CO_2_ molecule to the electro-generated 2Fe^II^ species inside the cofacial porphyrin cavity within the time scale of CV. The dissociation of the Cl^−^ and coordination of CO_2_ is supported by the UV-vis spectra of the chemically reduced *o*-Fe_2_^II^DTPP that shows the remarkable change of its Soret and Q-bands upon purging of CO_2_ gas at −30 °C, indicating the binding of CO_2_ to the Fe^II^ porphyrin species^49^. Indeed, the ability of CO_2_ to coordinate transition metal complexes is extensively investigated^51^. However, the coordination of CO_2_ to the electro-generated Fe^II^ species is not observed in the corresponding Fe porphyrin monomer under similar experimental conditions. Upon further scanning to more negative potential the electro-generated CO_2_-coordinated 2Fe^II^ species showed a reversible 2e^−^ redox couple corresponding to the generation of CO_2_ coordinated 2Fe^II^/2Fe^I^ species. The most interesting finding is the observation of a strong catalytic current in the presence of CO_2_ indicating electro-catalytic reduction of CO_2_ promoted by the six Fe porphyrin dimers. The appearance of the catalytic peak over the Fe^I^Fe^I^/Fe^I^Fe^0^ redox couple under Ar indicates the starting of the catalytic process once the Fe^I^Fe^0^ porphyrin species is electrochemically generated^49^. In general, the Fe porphyrin dimer with electron-withdrawing substituents shows electro-catalytic CO_2_ reduction behavior at more positive potential, i.e. at low *η*. In other words, the Fe porphyrin dimers are arranged in the following order with respect to their *η* (small to large) for the CO_2_ to CO conversion; Fe_2_DTPFPP < Fe_2_TPFPP-TMP < Fe_2_DTF_2_PP < Fe_2_DTCl_2_PP < Fe_2_DTPP < Fe_2_DTMP.

The effect of H_2_O content on the catalytic CO_2_ to CO conversion with Fe porphyrin dimers is tested by CVs at a 100 mV/s scan rate. Figure S3 (SI) shows the CVs of *o*-Fe_2_DTPP (0.5 mM) as a representative example at 100 mV/s scan rate in DMF containing 0.1 M ^*n*^But_4_NPF_6_ supporting electrolyte in the presence of different amounts of H_2_O under Ar or CO_2_. All the other Fe porphyrin dimers show similar behaviors. In H_2_O-free DMF solution, a large catalytic current was generated in a CO_2_-saturated solution. Increasing the H_2_O content to 10% in the medium induces the positive shift of the starting potential of the catalytic current and increases the catalytic peak current, Further addition of H_2_O above 10% decreases the catalytic peak current due to the decreasing solubility of the Fe porphyrin dimers.

For fast catalytic process, the foot-of-the-wave analysis of the CVs has been reported to be a quick estimation of the catalysis rate constant, *k*_*cat*_, TON, and TOF of the catalysis reaction without the contribution of side phenomena such as substrate consumption, catalyst deactivation, and/or product inhibition^19^,^23^. The analysis is based on the linear correlation between *i*/*i*^0^_p_ and 1/{1 + exp[*F*/*RT*(*E* − *E*^0^_cat_)]} “equation (7)”,where *i* is the catalytic current in the presence of CO_2_, *i*^0^_p_ is the peak current in the absence of CO_2_, *F*, *R*, *T*, and *E* are the Faraday constant, gas constant, absolute temperature, and the electrode potential, respectively. Plotting *i*/*i*^0^_p_ *vs.* 1/{1 + exp[*F*/*RT*(*E* − *E*^0^_cat_)]} gives rise to a straight line of slope 2.24(*RT*/*Fν*)^1/2^(*k*_*cat*_)^1/2^ (*ν* is the scan rate in V/s) from which the catalysis rate constant, *k*_*cat*_ is calculated. The *k*_*cat*_is then used to calculate the TOF and the logTOF −*η* relationship according to equations (8) and (9), respectively, where TOF^0^ is the intrinsic turnover frequency (turnover frequency at zero *η*). The value of *η* is calculated according to equation (10) based on the reported thermodynamic redox potential of the CO_2_ to CO conversion in DMF/5% H_2_O solution containing 0.1 M ^*n*^But_4_NPF_6_ supporting electrolyte, *E*^0^(CO_2_/CO) = −0.69 V^19^. Figure 3 depicts the catalytic CVs responses (left) of three Fe porphyrin dimers (0.5 mM, 100 mV/s scan rate) and the corresponding foot-of-the-wave analysis (right). Other catalysts show similar behaviors. Figure 4 shows the catalytic CVs responses (a) of the six Fe porphyrin dimers and the corresponding logTOF −*η* relationship (b). Table 1 summarizes the catalysis parameters of the current Fe porphyrin dimers and that of the most efficient reported CO_2_/CO reduction molecular catalysts. The table clearly shows that *η* for the CO_2_ reduction decrease by increasing the electron-withdrawing substituents on the porphyrin rings for the Fe porphyrin dimers and their benchmarking superiority for the CO_2_ to CO conversion activity over the reported catalysts.


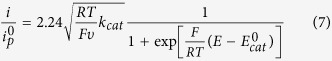



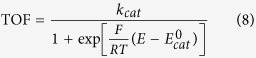



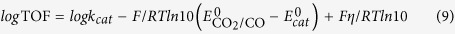



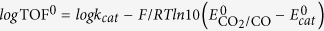



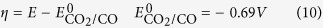


To test the activity, stability, and selectivity of the Fe porphyrin dimers for the CO_2_ reduction, a bulk electrolysis experiment was conducted in a gas-tight H shaped two-compartment electrochemical cell with a glass frit to separate the two compartments. The cell was filled with DMF/10% H_2_O solution (14 mL) containing 0.5 mM of the Fe porphyrin dimer and 0.1 M ^*n*^But_4_NPF_6_ as a supporting electrolyte. In one compartment, a glassy carbon working electrode (1 cm^2^) and a Ag/AgCl (3M NaCl) reference one were immersed close to each other (about 2 mm apart) in the solution. In the other compartment, a Pt foil (about 20 cm^2^) was immersed in the solution and used as a counter electrode. The solution in the two compartments was saturated with CO_2_ by bubbling CO_2_ for 30 min prior to the reaction. The products of the electro-catalytic reduction in the gas phase and in the solution phase were analyzed by gas chromatography and capillary electrophoreses, respectively. Figure 5a shows the current density-time profile of the electrolysis at −1.25 V *vs.* NHE (*η* = 0.56 V) in the presence and absence of the Fe_2_DTPFPP (0.5 mM) as a representative example of the Fe porphyrin dimers. The product analysis (Fig. 5b) of the headspace gas and solution shows the formation of CO gas in 92% Faradaic efficiency and H_2_ gas in 8% Faradaic efficiency. Only a very small amount of HCO_2_H detected in the solution, that means the Fe_2_DTPFPP dimer shows a high selectivity for CO_2_ reduction to CO. The other Fe porphyrin dimers show similar behavior for the CO_2_ to CO conversion however under slightly higher *η*. Based on the bulk electrolysis experiment, the catalysis parameters, *k*_*cat*_, TON, and TOF were calculated using equations (11)~(13);


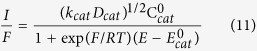



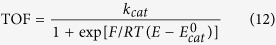



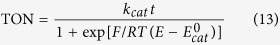


where, *I* is the net current density (the current density obtained from the bulk electrolysis corrected for the background current density and Faradic efficiency), *D*_cat_ is the diffusion coefficient of the catalyst calculated based on Randles-Sevcik equation^50^, and C^0^_cat_ is the bulk catalyst concentration, 5 × 10^−7^ mole/cm^3^ (0.5 mM). Table S2 (SI) summarizes the catalysis parameters of the six Fe porphyrin dimers obtained from the bulk electrolysis experiment conducted for 6 hrs in DMF/10% H_2_O saturated with CO_2_. The results of the bulk electrolysis experiments conducted at a single potential are presented as stars in Fig. 4b that demonstrate the validity of the foot-of-the-wave analysis for estimating the catalysis parameters^19^.

**Figure 1:**
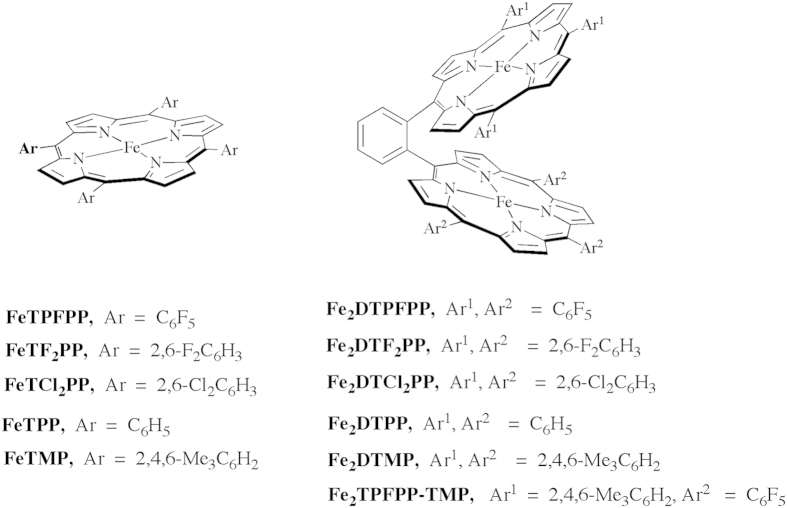
Chemical structures of the six Fe porphyrin dimers and their corresponding monomers.

To clarify the indispensableness of the dimer structure for the high catalytic activity as CO_2_ to CO conversion catalysts as well as to compare the catalytic performance between the dimers and the monomers, we prepared the corresponding Fe porphyrin monomers, FeTPFPP, FeTF_2_PP, FeTCl_2_PP, FeTPP and FeTMP (Fig. 1) as controls and measured their CV behaviors under Ar or CO_2_ in DMF/10% H_2_O solution. To normalize for the concentration of their iron centers, the Fe porphyrin monomers and dimers were tested at 1 mM and 0.5 mM concentration, respectively. Under Ar (Figure S2, SI), the Fe porphyrin monomers show three reversible 1e^−^ redox couples assigned for the Fe^III/II^, Fe^II/I^, and Fe^I/0^ at standard redox potentials depicted in Table S1. Under CO_2_, the Fe porphyrin monomers show catalytic current peaks for the CO_2_ reduction, however, lower than those shown by the corresponding Fe porphyrin dimers. This indicates the importance of the binuclear Fe centers for the high activity of the current Fe porphyrin dimers as CO_2_ to CO conversion catalysts. Figure S4 (SI) shows the CVs of the Fe_2_DTPFPP and FeTPFPP under Ar and CO_2_ as, a representative example, which clearly shows the high activity of the Fe porphyrin dimer compared to the corresponding monomer for CO_2_ to CO conversion without use of any acids.

For a clear kinetic comparison between the Fe porphyrin dimers and their corresponding monomers for CO_2_ to CO conversion, the catalytic peak current, *i* is normalized to the peak current of the Fe^II/I^, *i*^0^_p_. Figure S5 (SI) compares the activity of the six Fe porphyrin dimers with their corresponding monomers. Table S3 (SI) summarizes the catalysis parameters of the Fe porphyrin monomers, derived from the foot-of-the-wave analysis of the CVs. These results clearly elucidate that the binuclear structure is essential for highly efficient CO_2_ reduction. We tentatively propose that the cofacial dimer structure will provide a suitable way for the cooperation of the two Fe centers during the CO_2_ electro-catalytic reduction similar to that observed in CODHs. Consistent with this, the 1,3-phenylene bridged Fe porphyrin dimer, *m*-Fe_2_DTPP that has a large Fe-Fe separation distance (≈10.9 Å) showed very low activity (Table S3) for CO_2_ to CO conversion compared with the current cofacial Fe porphyrin dimers under the same conditions^49^.

## Discussion

In biological systems, CODHs use a [NiFe_4_S_4_] cluster, termed cluster C, to reversibly reduce CO_2_ to CO with high TON and TOF at low *η*. The crystallographic studies, at the atomic resolution (≤1.1 Å) of CODH in complex with CO_2_ revealed a CO_2_ ligand bridged between Ni and Fe in a μ_2_*η*^2^ coordination geometry that clearly indicates the cooperation between the binuclear Ni and Fe centers in the activation of CO_2_ reduction^36^. The presence of binuclear Ni and Fe centers in CODHs inspired us to utilize molecular inorganic catalysts containing binuclear centers for CO_2_ to CO conversion keeping in mind that the binuclear centers should be at a suitable separation distance to allow the cooperation between them. We previously utilized several cofacial porphyrin dimers as suitable ligands for holding two Mn ions at suitable Mn−Mn separation distances (3.7–6.2 Å) to promote water oxidation to O_2_ or H_2_O_2_ disproportionation^52^,^53^,^54^. As a first generation of molecular catalysts containing binuclear centers, we utilized six cofacial porphyrin dimers ligands with different electron donating and withdrawing substituents at the peripheral porphyrin rings and holding two Fe ions as bio-inspired catalysts for CO_2_ to CO conversion. Assuming similar Fe−Fe separation distances in the current catalysts, this will fit CO_2_ bridging to the Fe ions and allowing their cooperation for the CO_2_ to CO conversion. The results obtained demonstrate that, the Fe porphyrin dimers have the greatest activity, stability, and selectivity for CO_2_ electro-catalytic reduction to CO among the reported precious and non-precious CO_2_ to CO reduction molecular catalysts (Table 1). Control experiments with Fe porphyrin monomers (Figure S2, Table S3) and a 1,3-phenylene bridged Fe porphyrin dimer (Table S3) indicate the importance of the binuclear Fe centers and the Fe−Fe separation distance for the high catalytic performance.

**Figure 2:**
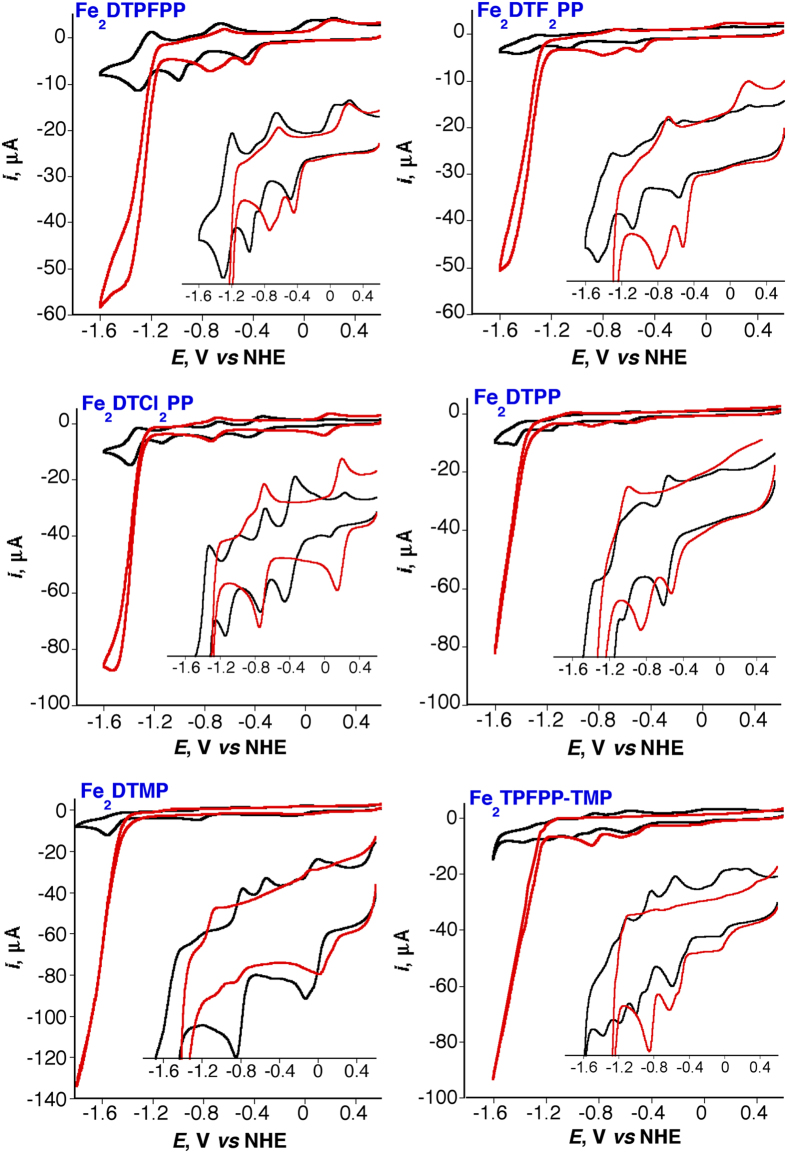
Cyclic voltammograms of the six Fe-porphyrin dimers (0.5 mM) in DMF/10% H_2_O at 50 mV/s scan rate under Ar (black lines) and CO_2_ (red lines). Insets: magnified traces of CVs.

**Figure 3:**
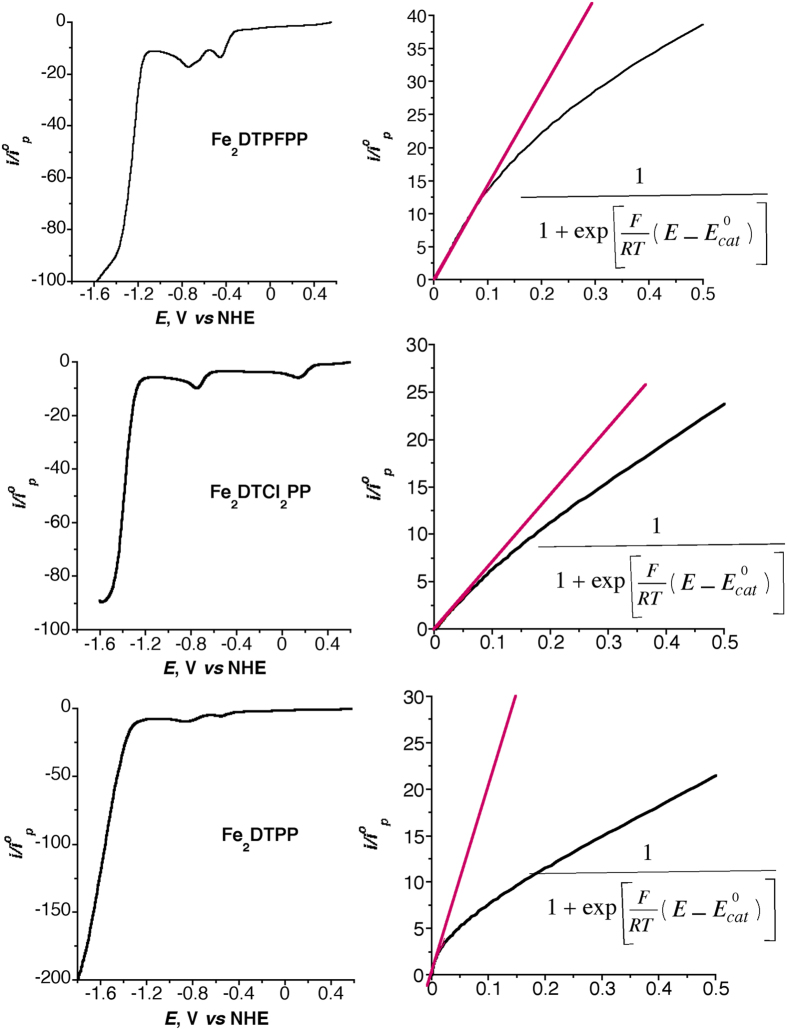
Catalytic CV responses (left, forward scan only shown for clarity) of three Fe porphyrin dimers (0.5 mM) at 100 mV/s scan rate in DMF/10% H_2_O saturated with CO_2_ and the corresponding foot-of-the-wave analysis (right).

On the time scale of CV, the data show that the cofacial Fe porphyrin dimers under Ar are reduced stepwisely as in equations (1)~(6). Under CO_2_, the catalytic current for the CO_2_ to CO conversion is observed on the peaks corresponding to [Fe^I^Fe^I^] to [Fe^I^Fe^0^] and [Fe^I^Fe^0^] to [Fe^0^Fe^0^] conversions as in equations (5) and (6) (Fig. 2). This means the active species for the CO_2_ reduction is either [Fe^I^Fe^0^] or [Fe^0^Fe^0^] species. The differentiation between the two species is unclear in the five symmetrical Fe porphyrin dimers where the two Fe centers have similar electronic environments. The hetero-dimer, Fe_2_TPFPP-TMP that has quite different electronic environment around the two Fe centers, on the other hand, showed clear separation of the stepwise reductions of the Fe centers under Ar (Table S1) and showed only strong catalytic current over the [Fe^I^Fe^0^] species (Fig. 2) under CO_2_. This observation indicates that Fe^0^ and Fe^I^ possibly act as a Lewis base and a Lewis acid, respectively, in the catalysis process. Through such roles of the binuclear centers, these Fe porphyrin dimers would promote the CO_2_ reduction without any acids^49^. At its lower reduced state, Fe^II^Fe^II^, these catalysts can capture CO_2_, judging from the appearance of a new CV peak under CO_2_ atmosphere (Fig. 2)^49^. This association of substrate CO_2_ and the catalysts at the lower reduced state will contribute to the increase of TOF in the dimer catalysts, because it is ready to do the rapid reduction of the bound CO_2_ at the catalyst Fe^0^Fe^I^ state.

**Figure 4:**
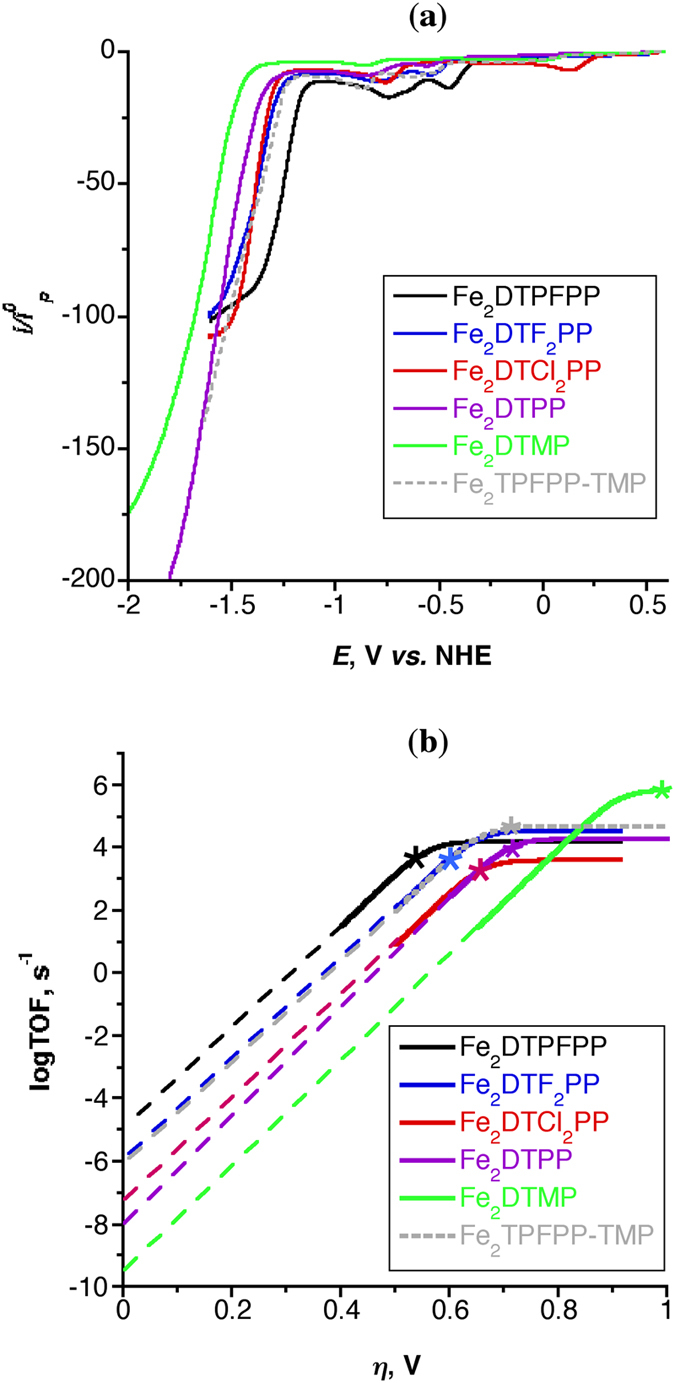
(**a**) Catalytic CVs responses (forward scan only shown for clarity) and (**b**) the Tafel plot [logTOF −*η* relationships] of the six Fe porphyrin dimers (0.5 mM) at 100 mV/s scan rate in DMF/10% H_2_O saturated with CO_2_. *logTOF resulted from bulk electrolysis experiments.

**Figure 5:**
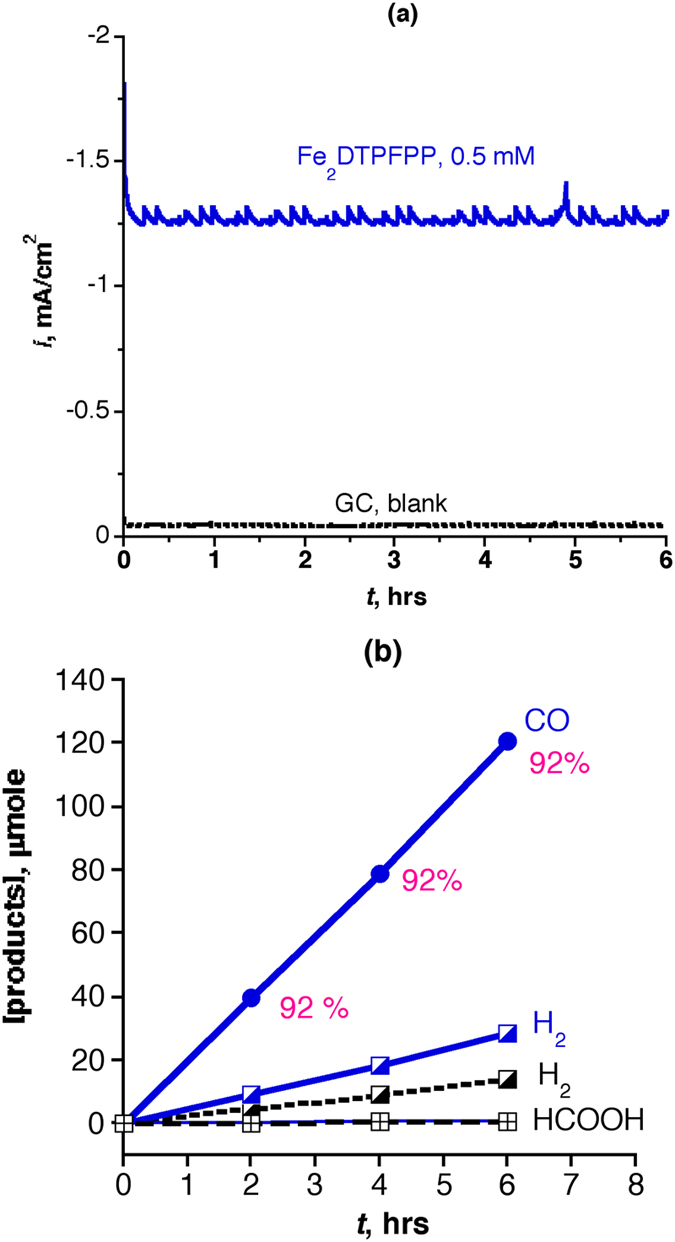
(**a**) Bulk electrolysis conducted for 6 hrs at −1.25 V *vs.* NHE (*η* = 0.56 V) and (**b**) products analysis of Fe_2_DTPFPP (0.5 mM) and GC blank in DMF/10% H_2_O under CO_2_.

The results obtained by the current cofacial Fe porphyrin dimers also indicate that *η* and TOF of the catalysis process can be tuned by the introduction of electronically different substituents to the porphyrin peripheral positions (Fig. 4 and Table 1). For example, introduction of electron-withdrawing perfluorophenyl substituents to the meso positions of the dimer reduces *η *≈ 0.3 V compared to that obtained with electron-donating mesityl group. In general, the introduction of electron-withdrawing groups on catalysts promotes the positive shifts of their reduction potentials, resultantly decrease of *η* of the reduction reaction. However, this electron-withdrawing effect also causes the decrease of the electron density at the active center, which sacrifices their nucleophilic activities to lead the decrease of TOF. On the other hand, electron-donating groups such as mesityl on the porphyrin rings leads high TOFs. In general, low reaction *η* and high TOF by electronic tuning is not compatible^22^, although TOFs for the dimer catalysts are kept within the moderate range even the catalysts bearing electron-withdrawing groups.

This work will turn the intention for designing new generations of highly efficient binuclear molecular catalysts for CO_2_ to CO conversion, working under neutral conditions. We are currently trying to obtain hetero-binuclear dimers to elucidate the catalytic mechanism.

## Methods

Materials, Instruments, CV measurements, Bulk electrolysis, and products analysis of CO_2_ reduction are presented in the supporting information.

### Preparation of Fe porphyrin dimers

The cofacial Fe porphyrin dimers were prepared as previously reported by stepwise procedures according to Scheme S1 (SI)^49^,^55^. We will discuss the preparation of [Fe_2_DTMP]Cl_2_ as a representative example in details. The other dimers were synthesized similarly.

### Preparation of 5-(2^′^-methylbenzoate)-10,15,20-trismesityl-porphyrin (compound b, Ar^1^ = 2,4,6-Me_3_C_6_H_2_)

A 2 L three-neck round-bottomed flask was charged with Methyl-2-formylbenzoate (**a**) (1.94 mL 11.8 mmol), mesitylaldehyde (4.5 mL, 36 mmol), pyrrole (3.2 mL, 48 mmol) and CHCl_3_ (600 mL). The colorless solution was purged with nitrogen for about 15 min. Then, boron trifluoride diethyl etherate (BF_3_·Et_2_O, 3.2 mL) was added via a syringe, accordingly the color of the solution changed immediately to dark red. The mixture was stirred at room temperature under nitrogen and TLC used to monitor the reaction progress. At the end of 5-hrs. reaction, DDQ (DDQ = 2,3-dichloro-5,6-dicyano-*p*-benzoquinone) solution (8 g/150 mL benzene, 36 mmol) was added to the reaction mixture, whose color changed to dark green. The mixture was stirred at room temperature for 1.5 hours. After solvent removal, the residue was dissolved into a small amount of CHCl_3_ and loaded into Al_2_O_3_ column then eluted with CHCl_3_. The first broad reddish violet band was collected. The TLC of this band showed the presence of three bands; a light red band on the top followed by strong reddish violet band then green and black bands on the bottom. The mixture was separated by a silica-gel column. The first red band eluted with *n*-hexane-benzene mixture (1:1 v/v) was collected and characterized as 0.2 g TMPH_2_ byproduct. The second reddish violet band contained 1.6 g of the desired compound b. The compound was further purified with a silica-gel column eluted with *n*-haxane- CHCl_3_ (1:1 v/v) to give the pure one (1.4 g, 14.8% yield). ^1^H NMR (CDCl_3_, 400 MHz): δ 8.65 (d, 8H, pyrrole β-H), 8.39 (m, 1H, Ar-H), 8.09 (m, 1H, Ar-H), 7.85 (m, 2H, Ar-H), 2.86 (s, 3H, OCH_3_), 2.64 (s, 9H, Ar-CH_3_), 2.00 (s, 3H, Ar-CH_3_), 1.88 (s, 3H, Ar-CH_3_), 1.86 (s, 9H, Ar-CH_3_), 1.80 (s, 3H, Ar-CH_3_), −2.46 (s, 2H, pyrrole N-H). MALDI-TOF-MS *m/z* = 798.25 (found), 799.02 (calcd.).

### Preparation of 5-(2^′^-Benzomethanol)-10,15,20-mesitylporphyrin (compound c)

A 500 mL flask was charged with compound **b** (0.5 g, 0.626 mmol) and dry THF (15 mL). The solution was cooled to 0 °C then LDBBA (LDBBA = lithium diisobutyl-*t*-butoxyaluminum hydride) reducing agent (12.5 ml, 4 mmol, 0.33 M solution) was added drop-wise. The reaction mixture was stirred for 3 hrs at 0 °C under N_2_ while its progress was monitored with TLC. After completed, the reaction quenched by adding 2M HCl and the product was extracted three times with CH_2_Cl_2_. The solvent was removed and the residue was purified with a silica-gel column using CH_2_Cl_2_ as an eluent to give a pure violet powder of the desired compound (0.47 g, 97.5% yield). ^1^HNMR (CDCl_3_, 400 MHz): δ 8.62 (d, 8H, pyrrole β-H), 8.02 (m, 1H, Ar-H), 7.93 (m, 1H, Ar-H), 7.82 (m, 1H, Ar-H), 7.63 (m, 1H, Ar-H), 4.38 (s, 2H, -CH_2_OH), 4.05 (s, 1H, -OH), 2.57 (s, 9H, Ar-CH_3_), 2.04 (s, 3H, Ar-CH_3_), 1.96 (s, 3H, Ar-CH_3_), 1.84 (s, 9H, Ar-CH_3_), 1.53 (s, 3H, Ar-CH_3_), −2.47 (s, 2H, pyrrole N-H). MALDI-TOF-MS *m/z* = 771.1 (found), 771.0 (calcd.).

### Preparation of 5-(2^′^-Benzaldehyde)-10,15,20-trismesityl-porphyrin (compound d)

Compound c (3.5 g, 4.5 mol) was dissolved into dry CH_2_Cl_2_ (500 mL) then excess active MnO_2_ (~15 g) was added. The solution was stirred under N_2_ for 2 hrs. The TLC showed complete conversion of the compound to d. The MnO_2_ was isolated by filtration. The solvent was removed under vacuum and the residue was purified with Silica-gel column using CH_2_Cl_2_ as eluent to give a pure violet powder of the desired compound (3.2 g, 91.6% yield). ^1^H NMR (CDCl_3_, 400 MHz): δ 8.66 (d, 8H, pyrrole β-H), 8.41 (m, 1H, Ar-H), 8.20 (m, 1H, Ar-H), 7.91 (m, 2H, Ar-H), 5.3 (s, 1H, -CHO), 2.62 (s, 9H, Ar-CH_3_), 1.87 (s, 18H, Ar-CH_3_), −2.52 (s, 2H, pyrrole N-H). MALDI-TOF-MS *m/z* = 768.5 (found), 768.99 (calcd.).

### Preparation of 1^′^,2^′^-bis[10,15,20-trimesitylporphyrin]-benzene (compound e)

A 1L three-necked flask was charged with compound d (2.0 g, 2.6 mmol), pyrrole (1.4 g, 20.8 mmol), mesitylaldehyde (3.05 g, 15.5 mmol), and dry CH_2_Cl_2_ (400 mL). The solution was stirred at room temperature for 30 min. under N_2_. Then, BF_3_·Et_2_O catalyst (5 mL) was added. After 4-hrs stirring, DDQ solution (5 g in 100 mL benzene) was added and the stirring is continued for 2 more hrs. The solvent was reduced by the rotary evaporator and loaded to Al_2_O_3_ column and the porphyrins mixture was collected using CHCl_3_ as eluent. The porphyrins mixture (TMPH_2_ and the dimmer) was separated by using silica-gel column eluted with CHCl_3_:hexane (1:3 v/v). The TMPH_2_ came first then the desired compound. The compound **e** was isolated as pure violet powder (0.85 g, 21% yield). ^1^H NMR (CDCl_3_, 400 MHz): δ 9.04 (d, 4H, pyrrole β-H), 8.73 (dd, 2H, Ar-H), 8.19 (d, 4H, pyrrole β-H), 8.19–8.15 (m, 2H, Ar-H), 8.11 (d, 4H, pyrrole β-H), 7.93 (d, 4H, pyrrole β-H), 7.02 (s, 2H, Ar-H), 7.01 (s, 4H, Ar-H), 6.99 (s, 2H, Ar-H), 6.76 (s, 4H, Ar-H), 2.55 (s, 18H, Ar-CH_3_), 1.47 (s, 36H, Ar-CH_3_), −3.52 (s, 4H, por-pyrrole NH). MALDI-TOF-MS *m/z* = 1547.2 (found), 1547.46 (calcd.).

### Preparation of diiron 1^′^-[10,15,20-trispentafluorophenyl-porphyrin]-2^′^-[10,15,20-trismesitylporphyrin]-benzene (Fe_2_DTMP, Compound f)

A solution of compound e (200 mg, 0.13 mmol) in dry DMF (20 mL) was refluxed overnight with excess FeBr_2_ (150 mg, 0.69 mmol). The solvent was then removed and the desired compound was extracted with CHCl_3_/1M HCl three times then with CHCl_3_/NaHCO_3_ (saturated) in three times and finally with the CHCl_3_/H_2_O in three times. The organic layer containing the desired compound was dried over anhydrous sodium sulfate. The compound was further purified with a silica-gel column using 5% MeOH-CHCl_3_ as an eluent. The reddish brown band was collected to give reddish brown powder (197 mg, 88.3% yield). MALDI-TOF-MS *m/z* = 1511, 1546, 1583 corresponding to Fe_2_DTMP (calcd 1511.5), [Fe_2_DTMP]Cl (calcd. 1546.95) and [Fe_2_DTMP]Cl_2_ (calcd. 1582.4), respectively.

Similarly, the other Fe porphyrin dimers were prepared and characterized by MALDI-TOF mass and UV-vis spectra. Fe_2_DTPFPP dimer shows peaks at *m/z* values of 1798.4 corresponding to Fe_2_DTPFPP (calcd. 1798.73) and 1834.0 corresponding to [Fe_2_DTPFPP]Cl (calcd. 1834.18). Fe_2_DTF_2_PP dimer shows peaks at *m/z* values of 1474.5 corresponding to Fe_2_DTF_2_PP (calcd. 1474.9). Fe_2_DTCl_2_PP dimer shows only one peak of *m/z* value of 1672.2 corresponding to Fe_2_DTCl_2_PP (calcd. 1672.36). The Fe_2_DTPP dimer shows peaks at *m/z* values of 1294.1 corresponding to [Fe_2_DTPP]Cl (calcd. 1294.47) and 1259.0 corresponding to Fe_2_DTPP (calcd. 1259.02). Fe_2_TPFPP-TMP shows peaks at *m/z* = 1690.1 and 1655.0 corresponding to [Fe_2_TPFPP-TMP]Cl (calcd. 1690.57) and Fe_2_(TPFPP-TMP) (calcd. 1655.1). Figure S1 (SI) depicts the MALDI-TOF mass spectra of the six Fe-porphyrin dimers and their UV-vis spectra (15 μM) in DMF.

## Additional Information

**How to cite this article**: Zahran, Z. N. *et al.* Bio-inspired cofacial Fe porphyrin dimers for efficient electrocatalytic CO_2_ to CO conversion: Overpotential tuning by substituents at the porphyrin rings. *Sci. Rep.* **6**, 24533; doi: 10.1038/srep24533 (2016).

## Supplementary Material

Supplementary Information

## Figures and Tables

**Table 1:** Catalysis parameters of Fe porphyrin dimers (this work) and other reported molecular CO_2_/CO reduction catalysts.

Solvent *E*^0^(CO_2_/CO), V	Catalyst *E*^0^_cat_ ,V	*k*_cat_, s^−1^	*η*, V	logTOF, s^−1^	logTOF^0^, s^−1^	Ref.
DMF/10% H_2_O −0.69	Fe_2_DTPFPP, −1.25	1.6 × 10^4^	0.40 − 0.60	1.5 − 3.9, 4.2*	−5.0	This work
DMF/10% H_2_O − 0.69	Fe_2_DTF_2_PP, −1.34	3.7 × 10^4^	0.50 − 0.65	2.0 − 4.3, 4.5*	−6.1	This work
DMF/10% H_2_O − 0.69	Fe_2_DTCl_2_PP, −1.35	4.1 × 10^3^	0.55 − 0.70	1.8 − 3.5, 3.6*	−7.2	This work
DMF/10% H_2_O − 0.69	Fe_2_DTPP, −1.40	2.0 × 10^4^	0.60 − 0.75	2.4 − 4.2, 4.3*	−8.1	This work^49^
DMF/10% H_2_O − 0.69	Fe_2_DTMP, −1.60	4.1 × 10^3^	0.70 − 0.85	2.3 − 4.8, 5.8*	−9.6	This work
DMF/10% H_2_O − 0.69	Fe_2_TPFPP, −TMP −1.35	7.3 × 10^5^	0.55 − 0.70	2.8 − 4.3, 4.7*	−6.1	This work
DMF/10% H_2_O − 0.69	FeTPP, − 1.41	2.1 × 10^3^	0.60 − 1.0	1.4 − 3.1, 3.3*	−8.4	This work
DMF/5% H_2_O /3M PhOH −0.69	CAT^a^, − 1.35	>5.0 × 10^6^	0.45 − 0.70	1.8 − 3.2, 3.8*	−6.0	22
DMF/5% H_2_O /3M PhOH −0.69	FCAT^b^, − 1.28	>5.0 × 10^6^	0.40 − 0.70	1.6 − 3.8, 4.0*	−5.5	22
DMF/5% H_2_O /3M PhOH −0.69	FeTPP, −1.43	3.5 × 10^4^	0.60 − 1.00	2.5 − 4.3, 4.5*	−8.0	20
CH_3_CN/0.8M CF_3_CH_2_OH −0.65	Re(bpy)(py)(CO)_3_ , −1.30	875.0	NA	2.9*	−8.0	11
CH_3_CN −0.65	(bbpy)Mn(CO)_3_ , −1.28	5.0 × 10^3^	NA	3.7*	−7.0	28
CH_3_CN −0.65	Ru(tpy)(Mebim-py), −1.34	59.0	NA	1.8*	−9.9	12
CH_3_CN −0.65	Ru(tpy)(bpy) −1.34	7.6	NA	0.9^*^	−10.8	12
DMF/0.1M HBF_4_ −0.23	*m*-(triphos)_2_Pd_2_ , −0.76	35.0	NA	1.5*	−7.4	42

^a^Fe *meso*-tetra(2,6-dihydroxyphenyl)porphyrin.

^b^Fe 5,15-di((2,6-dihydroxyphenyl)-10,20-di(pentafluorophenyl)porphyrin.

^*^logTOF_max_, s^−1^.
