# Supplementary material for: Bio-inspired cofacial Fe porphyrin dimers for efficient electrocatalytic CO2 to CO conversion: Overpotential tuning by substituents at the porphyrin rings
Source: Sci Rep. 2016 Apr 18;6:24533. doi: 10.1038/srep24533 (PMC4834491; doi:10.1038/srep24533)
Supplement: Supplementary Information [file srep24533-s1.doc]

**Supplementary Information**

**Bio-inspired cofacial Fe porphyrin dimers for efficient electrocatalytic CO_2_ to CO conversion. Overpotential tuning by substituents at the porphyrin rings.**

**Zaki N. Zahran,^1,2*^ Eman A. Mohamed,^1^ Yoshinori Naruta^1*^**

^1^Institute for Science and Technology Research, Centre for Chemical Energy Conversion, Chubu University, Kasugai 487-8501, Japan

^2^Faculty of Science, Tanta University, Tanta, Egypt

3

*Corresponding authors, znzahran@yahoo.com or naruta@isc.chubu.ac.jp

**Materials and Instruments.** Chemicals used in this study were purchased from Sigma-Aldrich Company and Wako Ltd, and were used without further purification. The lithium diisobutyl-*t*-butoxyaluminum hydride reductant (LDBBA) was prepared by literature methods.^S1^ Solvents were distilled from appropriate drying agents under nitrogen just prior to use. Mill-Q water (18 M Ω.cm) was used for solutions preparation. ^1^H NMR spectra were recorded on a JEOL JMX-GX 400 (400 MHz) spectrometer. Chemical shifts are reported as parts per million (ppm) with respect to CHCl_3_. Matrix Assisted Laser Desorption/Ionization Time-of-Flight (MALDI-TOF) mass spectra were measured on a BRUKER Auto flex II LRF20 spectrometer using dithranol as a matrix. UV-vis spectra were recorded using a Shimadzu UV-3100PC spectrophotometer. Electrochemical measurements were performed with a Metrohm Autolab Potentiostat/Galvanostat model PGSTAT 302. Gas chromatographic analyses were done with a Shimadzu GC-8A equipped with a capillary column (0.53 mm ID x 15 m) with a Molecular Sieve 5A layer at 40 ^o^C using Ar as a carrier gas. Capillary electrophoretic analyses were performed with Agilent model 7100 with use of a buffer (pH 6.0) consisting of quinolinic acid, hexadecyltrimethylammonium hydroxide, and 2- amino-2-hydroxymethyl-1,3-propandiol.

**Cyclic voltammetry measurements.** Cyclic voltammetric experiments were performed using a small three-electrode electrochemical cell. In a typical experiment, a total of 2.0 mL of DMF solution containing 0.1 M *^n^*But_4_NPF_6_ supporting electrolyte (*^n^*But_4_NPF_6_ = tetra-*n*-butylammonium hexafluorophosphate) and 0.5 mM of the dimer were used. A glassy carbon (3 mm ø), a Pt wire and a Ag/AgCl (3M NaCl) were used as a working, a counter, and a reference electrodes, respectively. All potentials reported in this work were referenced to normal hydrogen electrode (NHE) by addition of 0.198 V to the potential measured with respect to Ag/AgCl (3M NaCl) reference electrode. All measurements were made at room temperature 25°C.

**Reduction of carbon dioxide using controlled potential electrolysis.** The reduction of CO_2_ was carried out under controlled-potential electrolysis in a CO_2_ saturated DMF/10% H_2_O solution containing 0.1 M *^n^*But_4_NPF_6_ as supporting electrolyte. This was performed in a gas tight two-compartment cell: The cell was filled with DMF/10% H_2_O solution containing 0.1 M *^n^*But_4_NPF_6_ and 0.5 mM of the Fe porphyrin dimer. The solution was degased by Ar for 30 min, then saturated with CO_2_ gas by bubbling the gas for 30 min. A glassy carbon (1 cm^2^) and Ag/AgCl (3 M NaCl) working and reference electrodes were inserted close to each other and a Pt foil (20 cm^2^) was used as a counter electrode.

**Product analysis.** Calibration curves were constructed for CO and H_2_ gases detection as follow: The electrolysis cell was filled with the electrolysis solution (10 mL, DMF/10% H_2_O containing 0.1 M *^n^*But_4_NPF_6_), the solution was then purged with CO_2_ gas for 30 min then the cell was closed and different known amounts of CO and H_2_ gases were introduced to the headspace at different intervals (each 2.0 h) then 500 μl of the headspace gas was taken with a gas-tight syringe at a fixed interval and analyzed the samples on a GC. The areas of the H_2_ peak (at 0.9 min retention time) and that of CO (at 2.3 min retention time) were used for calibration of H_2_ and CO, respectively. The calibration curves for H_2_ and CO were then used to determine the generated CO and H_2_ gases produced during the bulk electrolysis experiments.

For HCO_2_H detection a calibration curve was constructed as follow: HCO_2_H standard solutions were prepared in the same solution (DMF/10% H_2_O) containing 0.1 M *^n^*But_4_NPF_6_, then 50 μl of the solution were transferred into 1 mL distilled water and a white precipitate of the supporting electrolyte was filtered off. The resultant clear solution was injected into a capillary electrophoresis instrument and eluted with buffer solution (pH 6.0). The area assigned for formic acid at 2.88 min retention time was used to make a calibration curve. The calibration curve was used to determine the concentration of the formic acid generated from the bulk electrolysis experiments.

**Table S1.** The standard redox potentials, *E*^0^, of the six Fe porphyrin dimers and their corresponding monomers in DMF/0.1 M *^n^*But_4_NPF_6_ under Ar. *E*^0^(1), *E*^0^(2), and *E*^0^(3) correspond to the simultaneous 2e^−^ reductions/oxidations of 2Fe^III/II^, 2Fe^II/1^ and 2Fe^I/0^ centers of the dimers and 1e^−^ reductions/oxidations of Fe^III/II^, Fe^II/1^ and Fe^I/0^ center of the monomers, respectively

| Catalyst | *E*^0^ (1) | | *E*^0^ (2) | | *E*^0^ (3) | | |
| --- | --- | --- | --- | --- | --- | --- | --- |
|  | *E*^0^ (1a) | *E*^0^ (1b) | *E*^0^ (2a) | *E*^0^ (2b) | *E*^0^ (3a) | *E*^0^ (3b) | |
| Fe_2_DTPFPP | −0.45 | | −0.78 | −0.95 | −1.25 | | |
| FeTPFPP | 0.23 | | −0.58 | | −1.08 | | |
| Fe_2_DTF_2_PP | −0.55 | | −0.87 | −1.05 | −1.34 | | −1.44 |
| FeTF_2_PP | 0.15 | | −0.67 | | −1.28 | | |
| Fe_2_DTCl_2_PP | −0.39 | | −0.72 | −1.07 | −1.35 | | |
| FeTCl_2_PP | −0.37 | | −0.67 | | −1.37 | | |
| Fe_2_DTPP | −0.59 | | −1.12 | | −1.40 | | −1.48 |
| FeTPP | 0.04 | | −0.78 | | −1.41 | | |
| Fe_2_TMP | −0.04 | −0.08 | −0.82 | | −1.60 | | |
| FeTMP | 0.03 | | −0.88 | | −1.61 | | |
| Fe_2_TMP-TPFPP | −0.45 | −0.59 | −0.87 | −0.91 | −1.35 | | −1.54 |

**Table S2.** The diffusion coefficients, *D*_cat_ (cm^2^/s), net current density, *I* (mA/cm^2^), *k*_cat_, and logTOF of the six Fe porphyrin dimers obtained from bulk electrolysis experiments conducted in DMF/10% H_2_O saturated with CO_2_ at overpotentials, *η*

| Catalyst | *D*_cat_ (cm^2^/s) | *η *(V) | *I* (mA/cm^2^) | *k*_cat_ | logTOF (s^-1^) |
| --- | --- | --- | --- | --- | --- |
| Fe_2_DTPFPP | 2.1 × 10^-7^ | 0.56 | 1.20 | 1.18 × 10^4^ | 3.8 |
| Fe_2_DTF_2_PP | 1.8 × 10^-8^ | 0.61 | 0.50 | 8.74 × 10^3^ | 3.8 |
| Fe_2_DTCl_2_PP | 7.0 × 10^-8^ | 0.66 | 0.45 | 4.97 × 10^3^ | 3.4 |
| Fe_2_DTPP | 8.1 × 10^-8^ | 0.71 | 1.00 | 2.12 × 10^4^ | 4.0 |
| Fe_2_DTMP | 1.4 × 10^-7^ | 1.06 | 0.80 | 2.36 × 10^8^ | 5.8 |
| Fe_2_TPFPP-TMP | 1.5 × 10^-7^ | 0.71 | 1.50 | 4.14 × 10^5^ | 4.7 |

Table S3. Catalysis parameters of CO_2_ to CO conversion of the Fe porphyrin monomers and 1,3-phenylene bridged Fe porphyrin dimer, *m*-Fe_2_DTPP, derived from the foot-of-the-wave analysis of CVs.

| **Solvent**  ***E*^0^(CO_2_/CO), V** | **Catalyst**  ***E*^0^_cat_, V** | ***k*_cat_, s^−1^** | ***η*, V** | **logTOF**  **s^−1^** | **logTOF^0^ s^−1^** |
| --- | --- | --- | --- | --- | --- |
| DMF/10% H_2_O  −0.69 | FeTPFPP  −1.08 | 8.3 | 0.40−0.60 | 0.7−0.9  .9* | −2.6 |
| DMF/10% H_2_O  −0.69 | FeTF_2_PP  −1.28 | 26.9 | 0.55−0.65 | 0.7−1.3  1.4* | −6.5 |
| DMF/10% H_2_O  −0.69 | FeTCl_2_PP  −1.37 | 111.8 | 0.56−0.70 | 0.1−1.9  2.0* | −8.8 |
| DMF/10% H_2_O  −0.69 | FeTPP  −1.41 | 2.1 x 10^3^ | 0.60−1.00 | 1.4−3.1  3.3* | −8.4 |
| DMF/10% H_2_O  −0.69 | FeTMP  −1.61 | 871.4 | 0.75−0.85 | 0.1−1.7  2.9* | −13.0 |
| DMF/10% H_2_O  −0.69 | *m*-Fe_2_DTPP  −1.39 | 1.3 x 10^3^ | 0.60−1.00 | 1.4−3.1  3.1* | −8.0 |

* logTOF_max_, s^−1^

**Scheme S1.** Synthetic route of the cofacial iron porphyrin dimers. Reaction conditions, **A**: pyrrole, arylaldehyde, BF_3_.Et_2_O, CHCl_3_, DDQ, **B**: LDBBA, **C**: MnO_2_, **D**: pyrrole, arylaldehyde, BF_3_.Et_2_O, CHCl_3_, DDQ, **E:** FeBr_2_, DMF

|  |  |
| --- | --- |
| **Figure S1.** Matrix Assisted Laser Desorption/Ionization Time-of-Flight (MALDI-TOF) mass spectra of the six Fe porphyrin dimers. Inset: UV-vis spectra of the six Fe porphyrin dimers (15 *μ*M) in DMF. | |

**Figure S2.** Cyclic voltammograms of the six Fe porphyrin dimers (0.5 mM) and their corresponding monomers (1.0 mM) in DMF/0.1 M *^n^*But_4_NPF_6_ at 50 mV/s scan rate under Ar.

**Figure S3.** CVs of Fe_2_DTPP (0.5 mM) as a representative example at 100 mV/s scan rate in DMF containing 0.1 M *^n^*But_4_NPF_6_ supporting electrolyte in the presence of different amounts of H_2_O under CO_2_.

**Figure S4.** CVs of Fe_2_DTPFPP dimer (top, 0.5 mM) and FeTPFPP monomer (bottom, 1mM) at 100mV/s scan rate at a glassy carbon working electrode in DMF/10% H_2_O under Ar (black lines) and CO_2_ (red lines).

**Figure S5.** *i/i^0^_p_* of the six Fe porphyrin dimers (0.5mM) and the corresponding Fe porphyrin monomers (1mM) in DMF/10% H_2_O at 100 mV/s scan rate under CO_2_.

**References**

# (S1) Kim, M. S., Choi, Y. M. & An, D. K. Lithium diisobutyl-**t**-butoxyaluminum hydride, a new and efficient reducing agent for the conversion of esters to aldehydes.

*Tettrahedron Letters* **48**, 5061-5064 (2007).
